# Supplementary material for: Protected areas reduce deforestation and degradation and enhance woody growth across African woodlands
Source: Commun Earth Environ. 2023 Oct 25;4(1):392. doi: 10.1038/s43247-023-01053-4 (PMC11041809; doi:10.1038/s43247-023-01053-4)
Supplement: Supplementary file 1 — Supplementary Information [file 43247_2023_1053_MOESM1_ESM.pdf]

Supplementary Information

Protected areas reduce deforestation and degradation and enhance woody growth across African woodlands

Iain M. McNicol[1], Aidan Keane[1], Neil D. Burgess[2, 3], Samuel J. Bowers[1], Edward T.A Mitchard[1], and Casey M. Ryan[1]

[1]School of GeoSciences, University of Edinburgh, Edinburgh, EH9 3FF, UK

[2]United Nations Environment Programme – World Conservation Monitoring Centre (UNEP-WCMC), Cambridge, CB3 0DL, UK

[3]Centre for Macroecology, Evolution and Climate, Natural History Museum, University of Copenhagen, Copenhagen, Denmark

Supplementary Methods

Covariates used for matching protected and unprotected areas

Based on the substantial empirical and theoretical work on the drivers of deforestation and degradation[1–6], we controlled for a set of variables widely considered to affect the probability of the outcomes in question. These include the suitability of the area for agriculture, its accessibility, extractable value and potential, and the geographic distance to markets and human populations. The data sources and methods used to create each layer are in Supplementary Table 1.

It is well established that the probability of deforestation (e.g. agricultural clearance) and degradation (e.g. woodfuel extraction) decreases with distance from human settlements and roads due to increasing difficulties accessing the forest resource, and the cost of transporting the product to markets[7,8]. Metrics of accessibility therefore include the estimated travel time (min) to the nearest city with a population greater than 50,000 in the year 2015[9], which accounts for the varying quality of road networks and the effect of local land cover and topography on travel speeds. This dataset is complemented by the geographic (Euclidean) distance (km) to both the nearest road[10], and the nearest settlement with a population <50,000. We also include the geographic distance to the nearest port, and/or cities with a population greater than 250,000 to capture both urban and international demand for resources.

In addition to accessibility, population density will also play a major role in determining whether an area is likely to be disturbed, and/or assigned protection, as it is likely easier to gazette areas where the local population is low, thus minimising competing interests for the land. As such, the population density in both 2005 and 2015[11] are included as separate covariates in our analysis to account for the baseline population pressures, and also any migration that may have occurred over the study period, which will likely result in changes in the demand for agricultural land, constructions materials, and wood-fuel.

Topographic variables including surface roughness, and the relative elevation of given pixel are also included as proxies of accessibility, with the former also including information on agricultural potential[12]. The relative elevation - defined as the difference in elevation between a pixel and its surrounding area – was included as it better describes small-scale accessibility compared to absolute elevation. We also combined data on topographic prominence with slope angle to generate a measure of broad topographic position (flat plain, slopes and hilltops), which is used an indirect proxy for agricultural suitability, and potential

biomass growth rates[13]. More direct, but relatively coarse resolution data on agricultural suitability for specific crops was extracted from the FAO–IIASA Project on Global Agro-Ecological Zones (GAEZ)[14] which combines information on climate, soil type, land cover, and topography to suitability maps for 49 major crop types, ranking each grid cell from 1 (very high suitability) to 8 (not suitable). We extracted the suitability maps for 11 crops that are among the most widely harvested in each country according to FAOSTAT, or are known to be major cash crops. Separate suitability maps were created for subsistence crops including Maize, Sorghum, Rice, Millet, Sweet Potato and Cassava, and the 'cash' crops of Groundnut, Sunflower, Soybean, Sesame, and Tobacco. We assume that any cultivation of these crops will be predominantly low input (i.e. no fertiliser or irrigation), which narrows the range of suitability. For each location, we extracted the highest suitability value from each set of crops as our measure of agricultural potential.

The final set of variables are those related to the extractable value of the woodlands and forests, which will be an important factor in determining the probability of degradation. Charcoal production, timber and construction material extraction all involve the selective logging of trees of a preferred size class and species[15,16]. In the absence of ground data, the percentage woody cover (25 m pixels >10 Mg C ha^{-1}), and average carbon density (Mg C ha^{-1}) at T1 within a 1 km cell are used as proxies for extractable value. This assumes areas of high woody cover and biomass may be targeted for timber extraction and charcoal production (i.e. degradation) as they are more likely to be contain trees of harvestable size. Controlling for both baseline woody cover and biomass also reduces the likelihood that a matched cell has already undergone widespread degradation and deforestation, and that both stand-age and species-specific differences in growth rates do not have undue influences on the relative change in AGC[2].

Unprotected control cells were selected from within the same country and political region – i.e. the administrative unit below national level ('region' in Tanzania, province in 'Mozambique'), and those regions adjacent to ensure that matches are close to PAs under investigation and so avoid matched areas with very different socio-economic, or climatic characteristics. These potentially hidden biases may include:

(i) Differences in forest governance between administrative units (e.g. the enforcement of forest protection)
(ii) Differences in the cost of inputs and commodity prices (outputs), such timber and agricultural products

(iii) The affluence of the local population and degree of dependence on natural resources

(iv) Climate variation (mean annual precipitation and seasonality) which may influence agricultural productivity and/or intensity[4,17,18] (Figure S2)

Supplementary Tables

Supplementary Table 1 - Description and sources for the matching covariates and the rationale for the selected bin sizes used for coarsened exact matching. The data was primarily rescaled by averaging all values within a 1 km^2 cell, or if the dataset was at a coarser resolution, by bilinear interpolation.

Matching covariate	Predicts	Source, attributes and bins for coarsened exact matching
**Travel time to major city (minutes)**	Degradation	The estimated travel time to the nearest city with a population >50,000 in the year 2015[9] . The dataset is provided at 1 km resolution. For the matching procedure, values were separated into bins of 60 minutes up to 240 minutes travel time, after which the data is aggregated into 120-minute bins.
**Distance to nearest road (km)**	Deforestation Degradation	Road networks were obtained from Open Street Map. The dataset was aggregated to 1 km resolution, after which the geographic distance to other cells was calculated. Studies show the probability of area being disturbed decreases 4 – 15 km from the road network and smaller settlements[8,19]. The data was therefore aggregated into bins of 0 - 2 km and 2 – 4 km, after which the data was placed in 4 km bins up to 26 km, after which we consider any change in the likelihood of disturbance to be minimal. The extension of the maximum distance at which disturbance is typically observed is to account for unmapped smaller tracks emanating from the road network.
**Distance to nearest settlement (km)**	Deforestation Degradation	Settlements with a population <50,000 in the year 2000, which are not included in the Travel Time layer, were obtained from Global Rural Urban Mapping Project (v4). This is supplemented by the ESRI World Cities dataset, which provides more up to date information on both the location and population of major administrative centres. For this layer, we use the same bin sizes as the roads dataset. The 'distance to nearest city' layer includes any city with a population >250,000, and any major port cities not meeting that criteria. For matching, we aggregated the data into 50 km bins up to 150km, after which a 100 km bin was used.
**Distance to nearest major city (km)**	Deforestation Degradation	
**Population density (persons sq km)**	Deforestation	The estimated population density in 2005 and 2015 were obtained Gridded Population of the World (GPW) v4. These are generally derived using district-level population counts, which are proportionally allocated to 1 km grid cells. In order to provide a continuous surface, and better account for surrounding population pressures, the average population density within a 50 km radius moving window was calculated, respecting national boundaries. The bins used for matching were; 0 - 50 (rural population); 50 - 100 (peri-urban); 100+ (urban).

<table>
<tr><td>Relative elevation (m)</td><td>Degradation</td><td rowspan="3">All three topographic variables are based on the 90m resolution Shuttle Radar Topographic Mission (SRTM) dataset[20]. The data was averaged to 1 km resolution to create a map of absolute elevation (m) from which we calculated the Relative Elevation (m), or relative prominence of a 1 km cell relative to its surroundings, as the vertical distance between a given pixel and the value of the lowest quantile of elevation inside a 50 x 50 km moving window. The second variable is Topographic Roughness which is standard deviation of the 90 m elevation data within a 5 x 5 pixel moving window (~1 km^2), with the resulting dataset then averaged to 1 km resolution.</td></tr>
<tr><td>Topographic roughness</td><td>Deforestation
Degradation</td></tr>
<tr><td>Topographic position</td><td>Deforestation</td></tr>
<tr><td>Crop suitability</td><td>Deforestation</td><td>Data on crop suitability was derived from the IFAO–IIASA Project on Global Agro-Ecological Zones (GAEZ)[14]. The data is provided at 10 km resolution with separate datasets created for subsistence crops and cash crops. The bins used for matching were: 1 - 2 (v. high – high suitability); 3 - 5 (medium - moderate); 6 - 7 (marginal - v. marginal); 8 (not suitable)</td></tr>
<tr><td>Wooded area (%)</td><td>Degradation</td><td>The percentage wooded cover in each 1 km cell at T1 was divided in to the following bins: 0 - 10% (non –wooded land); 10 - 25% (very sparse woodland); 25 - 50% (sparse woodland); 50 - 75% (woodland); 75+% (dense woodland)</td></tr>
<tr><td>Average AGC (Mg C ha^{-1})</td><td>Degradation</td><td>The average carbon density within a 1-km^2 cell in the period 2007 - 2010. Data were separated into bins of 0 – 15, 15 – 30, 30 - 50 and >50 Mg C ha^{-1}. These breakpoints in AGC storage tend to be associated with shifts in species composition and so may also account for differences in vegetation types[21]</td></tr>
</table>

Supplementary Figures

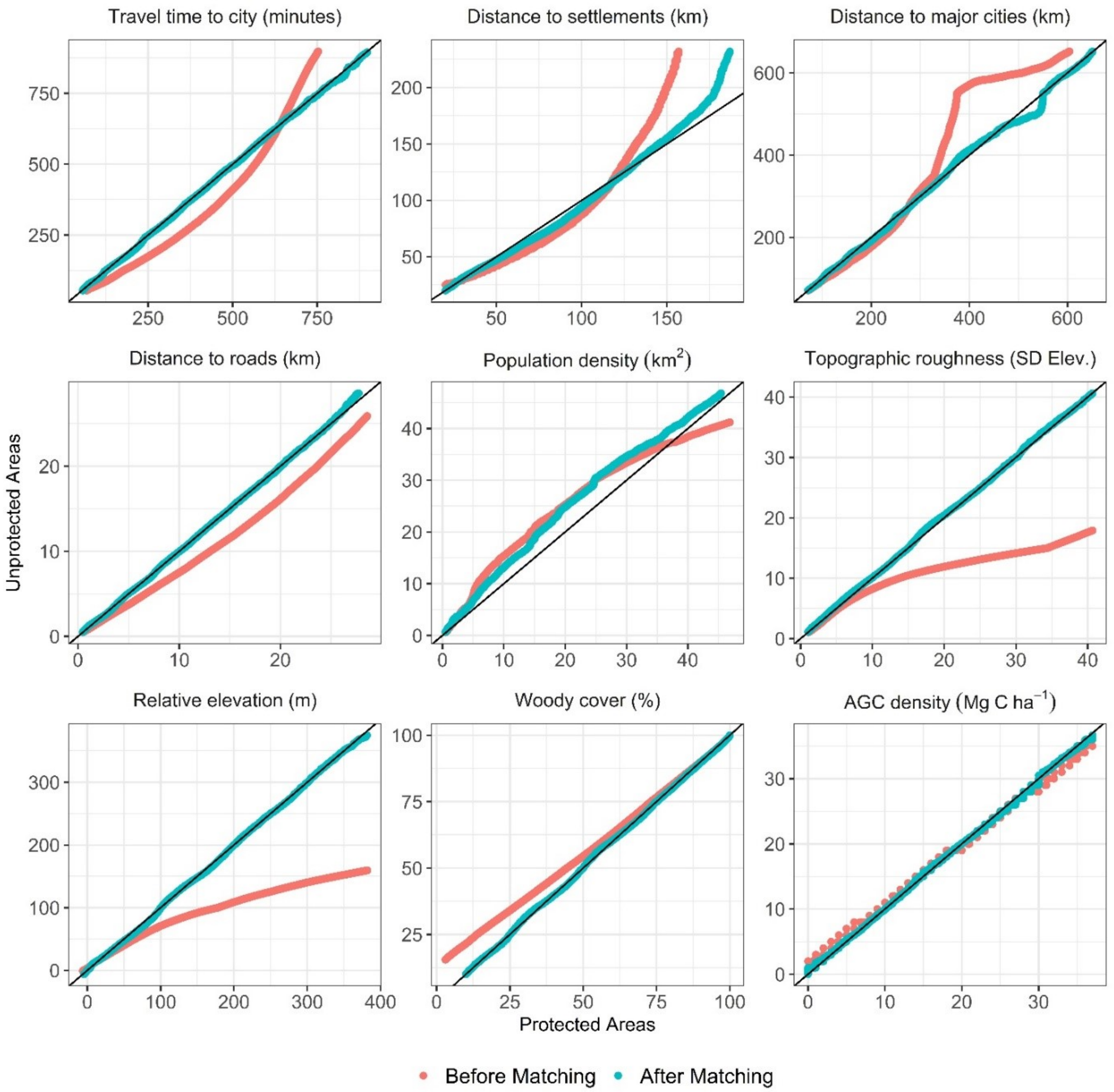

Supplementary Fig. 1 | Covariate balance before and after matching. Quantile-Quantile (QQ) plots are used to compare the distribution of the covariates inside and outside protected areas before and after matching. If the points lie along the 1:1 line then both the protected, and unprotected data have a similar distribution. If the points lie below the 1:1 line, then a larger proportion of PAs have those values than in unprotected areas.

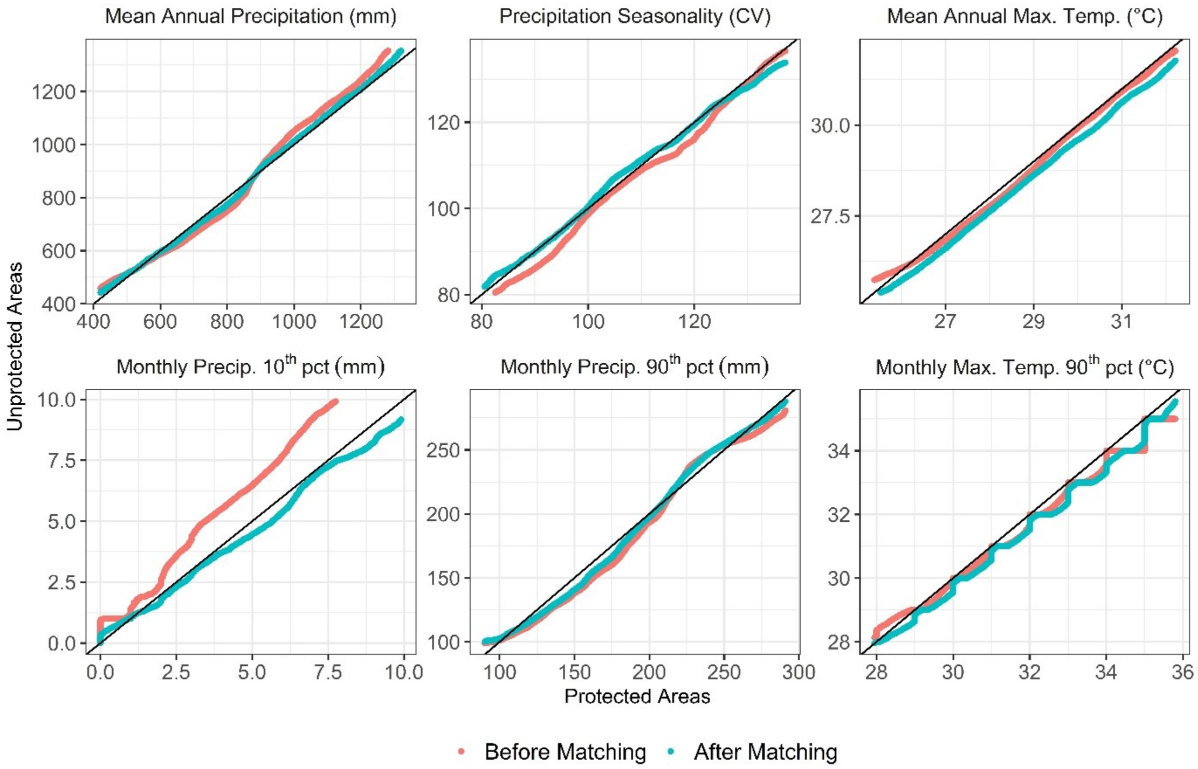

Supplementary Fig. 2 | Post-hoc comparison of the climate conditions in protected and unprotected areas before and after matching. As in Supplementary Fig. 1, Quantile-Quantile plots are used to compare the climate conditions in the pre- and post-matching samples to see whether this has an effect on the results. This was done using monthly estimates from WorldClim[22] covering the period 2010 - 2019 (last year excluded to fit with study period). This includes comparisons of the general climate conditions, including the mean annual total precipitation (mm/ year) and mean seasonality - the variation of rainfall within a single year, expressed by the coefficient of variation - and the mean annual maximum temperature. To capture extremes, the 10th and 90th percentiles of the monthly precipitation data across all years (*n* months = 108) are used as a proxy for drought and flooding respectively, and the 90th percentile of the monthly maximum temperature data as a proxy for heatwaves. The results of this show no meaningful difference in these variables between matched PAs and non-PAs, meaning our estimates of PA effectiveness are robust to climatic conditions

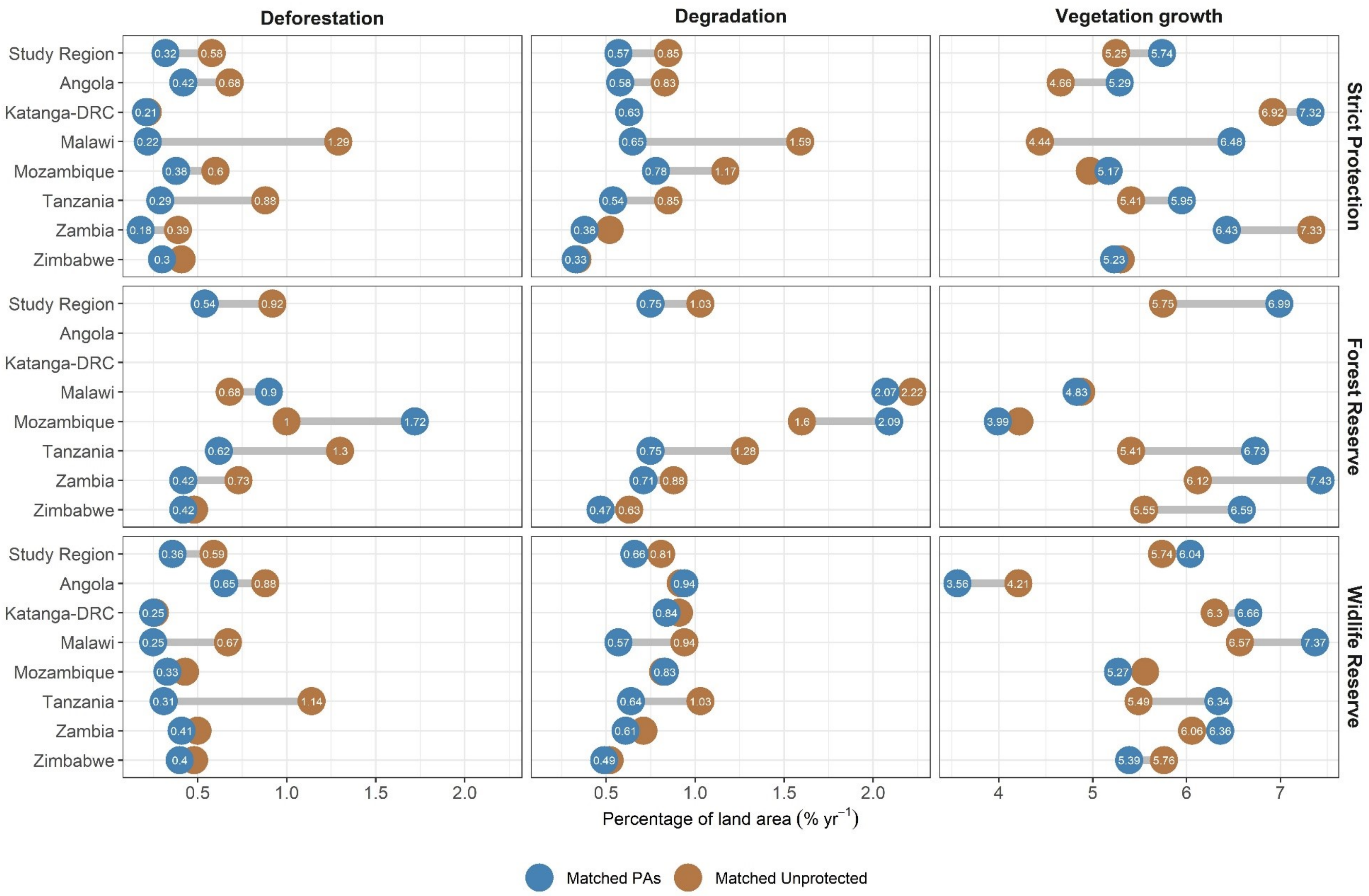

Supplementary Fig. 3 | Effect of protected area designation on land use and land cover changes within each country. Comparison of the deforestation, degradation and vegetation growth in protected and unprotected areas, separated by PA designation, and country. The 95% confidence intervals on each estimate can be calculated applying the following proportions to the reported values; Deforestation: 0.89 – 1.14; Degradation: 0.90 – 1.13, and Vegetation Growth: 0.92 – 1.09

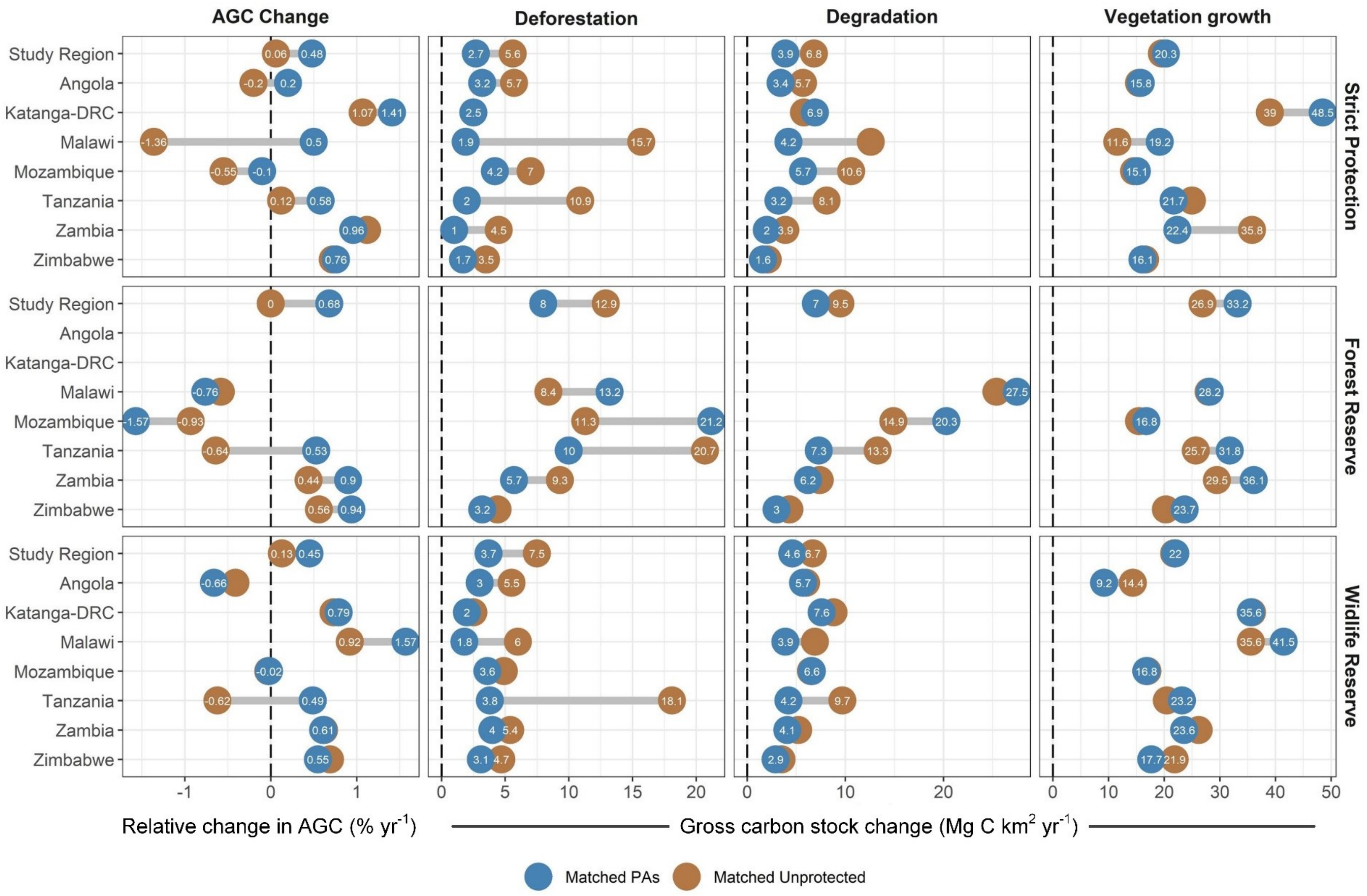

Supplementary Fig. 4 | Effect of protected area designation on carbon stock changes within each country. Comparison of the net AGC change, and the gross carbon stock changes due to deforestation, degradation and vegetation growth in protected and unprotected areas, separated by PA designation, and country. The 95% confidence intervals around the gross estimates of change are not included for stylistic reasons, however these can be calculated by applying the following proportions to the reported values; Deforestation: 0.86 – 1.15; Degradation: 0.88 – 1.11, and Vegetation Growth: 0.86 – 1.12.

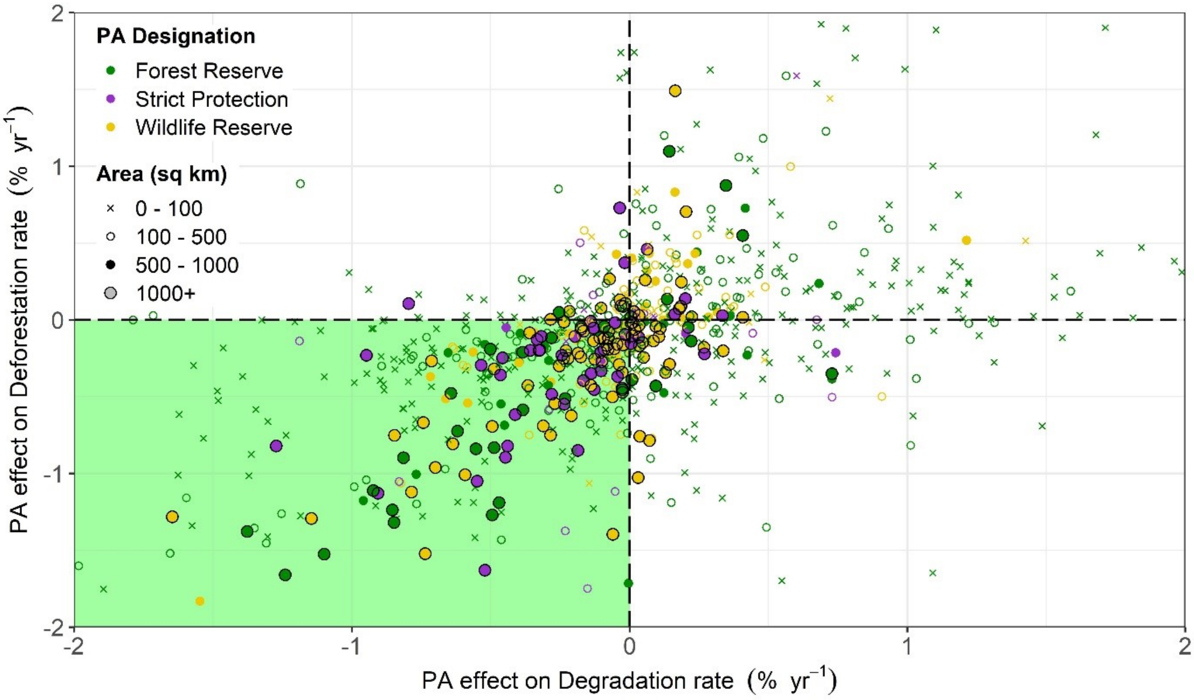

Supplementary Fig. 5 | Effectiveness of individual PAs on rates of deforestation and degradation. The effectiveness of PAs at reducing deforestation and degradation, calculated by comparing rates between matched protected and unprotected areas. The green shading shows PAs that reduce deforestation and degradation relative to their matched counterparts. The

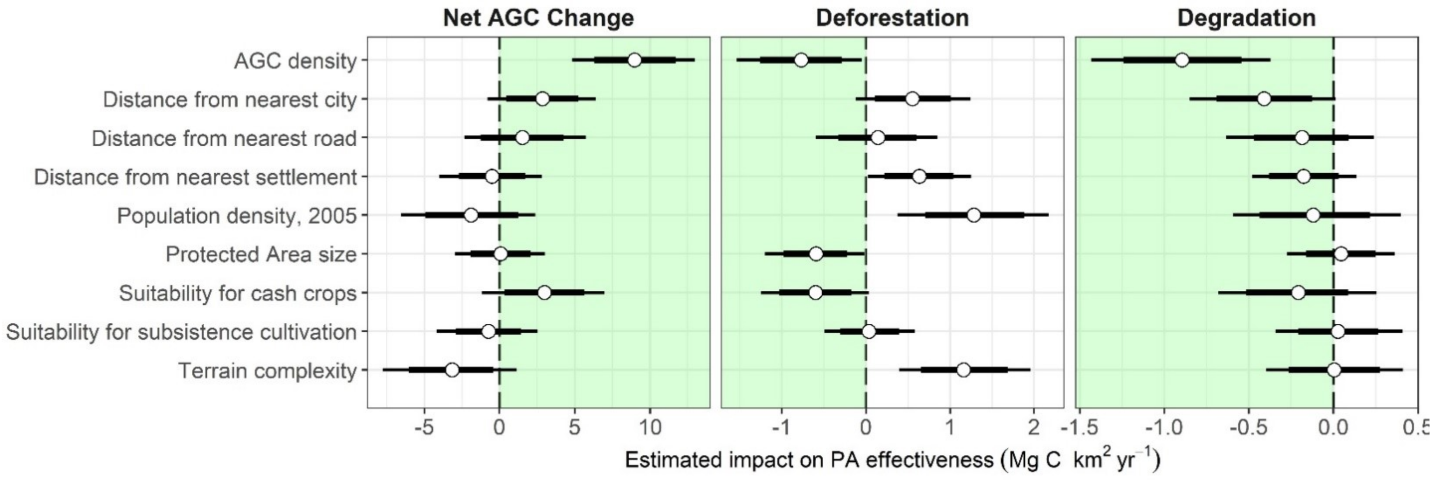

Supplementary Fig. 6 | Impact of covariates on PA effect score. The estimated impact of observable covariates used in the matching process on net ΔAGC, and gross carbon losses from deforestation and degradation. The circles indicate the mean estimate of the interaction effect, with thick lines showing the 80% credible intervals and thin lines show the 95% credible intervals around these mean values.

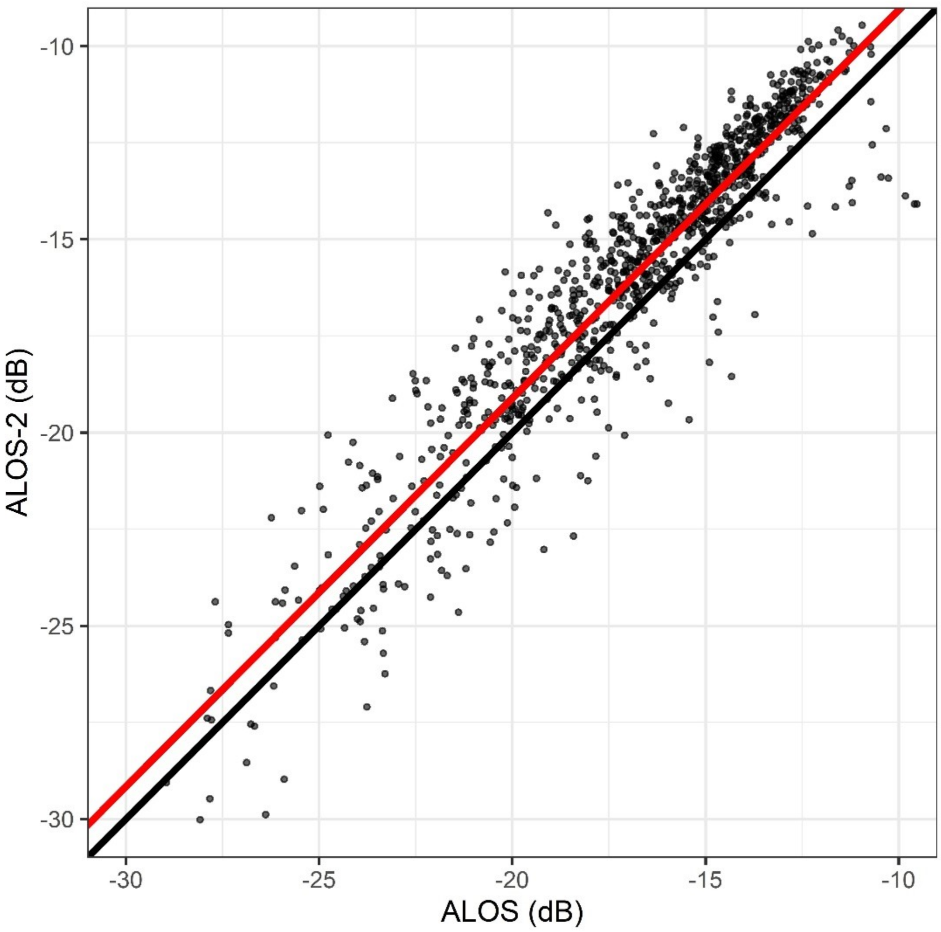

Supplementary Fig. 7 | ALOS2 - ALOS cross-calibration model. The model is used to bring ALOS-2 in line with the ALOS data and is developed using 1000 pseudo-invariant sites where no significant change is likely to have occurred over the study period.

Supplementary References

1. Ryan, C. M., Berry, N. J. & Joshi, N. Quantifying the causes of deforestation and degradation and creating transparent REDD+ baselines: A method and case study from central Mozambique. *Appl. Geogr.* **53**, 45–54 (2014).
2. McNicol, I. M., Ryan, C. M. & Williams, M. How resilient are African woodlands to disturbance from shifting cultivation? *Ecol. Appl.* **25**, 2330–2336 (2015).
3. Geist, H. J. & Lambin, E. F. *What Drives Tropical Deforestation?* (2002).
4. Angelsen, A. & Kaimowitz, D. Rethinking the causes of deforestation: lessons from economic models. *World Bank Res. Obs.* **14**, 73–98 (1999).
5. Rudel, T. K. The national determinants of deforestation in sub-Saharan Africa. *Philos. Trans. R. Soc. Lond. B. Biol. Sci.* **368**, 20120405 (2013).
6. Green, J. M. H. *et al.* Deforestation in an African biodiversity hotspot: Extent, variation and the effectiveness of protected areas. *Biol. Conserv.* **164**, 62–72 (2013).
7. Rideout, A. J. R., Joshi, N. P., Viergever, K. M., Huxham, M. & Briers, R. a. Making predictions of mangrove deforestation: a comparison of two methods in Kenya. *Glob. Change Biol.* **19**, 3493–3501 (2013).
8. Dons, K., Panduro, T. E., Bhattarai, S. & Smith-hall, C. Spatial patterns of subsistence extraction of forest products - an indirect approach for estimation of forest degradation in dry forest. *Appl. Geogr.* **55**, 292–299 (2014).
9. Weiss, D. J. *et al.* A global map of travel time to cities to assess inequalities in accessibility in 2015. *Nature* **553**, 333–336 (2018).
10. OpenStreetMap contributers. Planet dump retrieved from https://planet.osm.org. (2022).
11. CIESIN. Gridded Population of the World Version 4 (GPWv4): Population Density Grids. (2016).
12. Jansen, L. J. M., Bagnoli, M. & Focacci, M. Analysis of land-cover/use change dynamics in Manica Province in Mozambique in a period of transition (1990-2004). *For. Ecol. Manag.* **254**, 308–326 (2008).
13. Woollen, E., Ryan, C. M. & Williams, M. Carbon Stocks in an African Woodland Landscape: Spatial Distributions and Scales of Variation. *Ecosystems* **15**, 804–818 (2012).
14. FAO and IIASA. Global Agro-Ecological Zones (GAEZ v3).
15. Ahrends, A. *et al.* Predictable waves of sequential forest degradation and biodiversity loss spreading from an African city. *Proc. Natl. Acad. Sci. U. S. A.* **107**, 14556–14561 (2010).

16. Woollen, E. *et al.* Charcoal production in the Mopane woodlands of Mozambique: what are the tradeoffs with other ecosystem services? *Philos. Trans. R. Soc. B-Biol. Sci.* (2016).

17. Shah, P. & Baylis, K. Evaluating Heterogeneous Conservation Effects of Forest Protection in Indonesia. *Plos One* **10**, (2015).

18. Gaveau, D. L. a *et al.* Evaluating whether protected areas reduce tropical deforestation in Sumatra. *J. Biogeogr.* **36**, 2165–2175 (2009).

19. Wessels, K. J. *et al.* Unsustainable fuelwood extraction from South African savannas. *Environ. Res. Lett.* **8**, 014007 (2013).

20. Farr, T., Rosen, P. & Caro, E. The shuttle radar topography mission. *Rev. Geophys.* **45**, 1–33 (2007).

21. McNicol, I. M., Ryan, C. M., Dexter, K. G., Ball, S. M. J. & Williams, M. Aboveground carbon storage and its links to forest structure, tree species diversity and floristic composition in south-eastern Tanzania. *Ecosystems* **21**, 740–754 (2018).

22. Fick, S. E. & Hijmans, R. J. WorldClim 2: new 1-km spatial resolution climate surfaces for global land areas. *Int. J. Climatol.* **37**, 4302–4315 (2017).
