# Supplementary material for: Protected areas reduce deforestation and degradation and enhance woody growth across African woodlands
Source: Commun Earth Environ. 2023 Oct 25;4(1):392. doi: 10.1038/s43247-023-01053-4 (PMC11041809; doi:10.1038/s43247-023-01053-4)
Supplement: Supplementary file 2 — Description of Additional Supplementary Files [file 43247_2023_1053_MOESM2_ESM.pdf]

Description of Additional Supplementary Files

Protected areas reduce deforestation and degradation and enhance woody growth across African woodlands

Iain M. McNicol *et al*. (2023) *Communications Earth and Environment*

File name: Supplementary Data

Description of data:

Supplementary Data 1 / Supplementary Table 2

Change statistics for each country, region and district in our study region. All carbon stock and area change estimates are from areas are for wooded lands, i.e. areas with an AGC density ≥10 Mg C ha^{-1} at the start of the study period

Supplementary Data 2 / Supplementary Table 3

Change statistics for each Protected Area within our study region, based on the May 2021 version of the World Database of Protected Areas (WDPA). All carbon stock and area change estimates are from wooded lands, i.e. areas with an AGC density ≥10 Mg C ha^{-1} at the start of the study period. Estimates of carbon stocks and changes are expressed in metric tonnes (Mg). Our estimates of Protected Area impacts are in Supplementary Data 3.

Supplementary Data 3 / Supplementary Table 4

The average carbon stock and land cover change inside matched protected and unprotected (control) areas. The data were aggregated (summed) to 1km2 prior to analysis and are expressed on an annual basis given the different time-periods covered by the satellite radar data across the study region. The areas affected by each land cover processes are expressed as the average number hectares per 1 km^2 grid cell, which is analogous to the % of each grid cell. Carbon stock changes are expressed as the average number tonnes of carbon gained or lost per 1km^2 grid cell per year.
